# Negative impacts from latency masked by noise in simulated beamforming

**DOI:** 10.1371/journal.pone.0254119

**Published:** 2021-07-01

**Authors:** Jordan A. Drew, W. Owen Brimijoin

**Affiliations:** 1 Facebook AR/VR - Audio, Redmond, Washington, United States of America; 2 Department of Electrical and Computer Engineering, University of Washington, Seattle, Washington, United States of America; University of California, Los Angeles, UNITED STATES

## Abstract

Those experiencing hearing loss face severe challenges in perceiving speech in noisy situations such as a busy restaurant or cafe. There are many factors contributing to this deficit including decreased audibility, reduced frequency resolution, and decline in temporal synchrony across the auditory system. Some hearing assistive devices implement beamforming in which multiple microphones are used in combination to attenuate surrounding noise while the target speaker is left unattenuated. In increasingly challenging auditory environments, more complex beamforming algorithms are required, which increases the processing time needed to provide a useful signal-to-noise ratio of the target speech. This study investigated whether the benefits from signal enhancement from beamforming are outweighed by the negative impacts on perception from an increase in latency between the direct acoustic signal and the digitally enhanced signal. The hypothesis for this study is that an increase in latency between the two identical speech signals would decrease intelligibility of the speech signal. Using 3 gain / latency pairs from a beamforming simulation previously completed in lab, perceptual thresholds of SNR from a simulated use case were obtained from normal hearing participants. No significant differences were detected between the 3 conditions. When presented with 2 copies of the same speech signal presented at varying gain / latency pairs in a noisy environment, any negative intelligibility effects from latency are masked by the noise. These results allow for more lenient restrictions for limiting processing delays in hearing assistive devices.

## Introduction

While hearing assistive devices prove useful in quiet situations, such as one-on-one conversations in a quiet room or watching television, they can fail to provide a significant intelligibility benefit in everyday situations such as a busy cafe, restaurant, or street corner. Such scenes often contain multiple auditory objects (multiple talkers, music, traffic, etc.) that are competing for the auditory system’s attention. A degraded auditory system has trouble segregating these objects into distinct sources that the brain can interpret as a cohesive message, partly due to the reduced frequency resolution that accompanies sensorineural hearing loss [1], partly due to a decline in temporal synchrony [2], and at least in principle both contributing to a decline in spectrotemporal processing [3]. Furthermore, spatial hearing also tends to decline in association with presbycusis [4]. The summation of these deficits can make it extremely challenging for someone experiencing hearing loss to correctly perceive speech, especially in noisy environments, and there is little evidence suggesting that hearing aids can fully address these issues.

The healthy auditory system does a sufficient job in segregating auditory objects in a process called auditory scene analysis by grouping acoustic properties, e.g., overlapping frequency, timing of onset and offset, as well as direction of arrival, from a single auditory object over time [5]. For example, the brain can utilize spatial cues to perceptually segregate auditory objects that occupy separate locations in space as evidenced by spatial release from masking (SRM) [6]. Those impacted by hearing impairment are less able to use SRM [7]. There are two potential ways to assist the hearing impaired in taking advantage of auditory objects separation in space. The first would be to further spatially separate the signal of interest from the noise, but this is prohibitively difficult in everyday situations in which we have little control over our environment. The second would be to attenuate the level of the noise. Adaptive beamforming algorithms can be designed using linear FIR filters in order to increase signal-to-noise ratio (SNR) of the target speech, assumed to be in front of the listener, while attenuating surrounding noise. The length of the filter can be adjusted for the specific situation to attain a desired SNR. With increased filter length the greater the SNR can be achieved and the larger the processing delay becomes. In an open fit hearing aid the listener may receive acoustic information directly from the talker in addition to the reinforced but necessarily delayed signal from the hearing assistive device. Given that increased SNR comes at the cost of latency, the question becomes: is the benefit of the enhancement worth the impact of latency? In other words, are there negative impacts on intelligibility that outweigh the benefits provided by the beamformer? This study aimed to investigate how certain gain / latency pairs from simulated beamformers impact the intelligibility of speech in noise.

**Fig 1:**
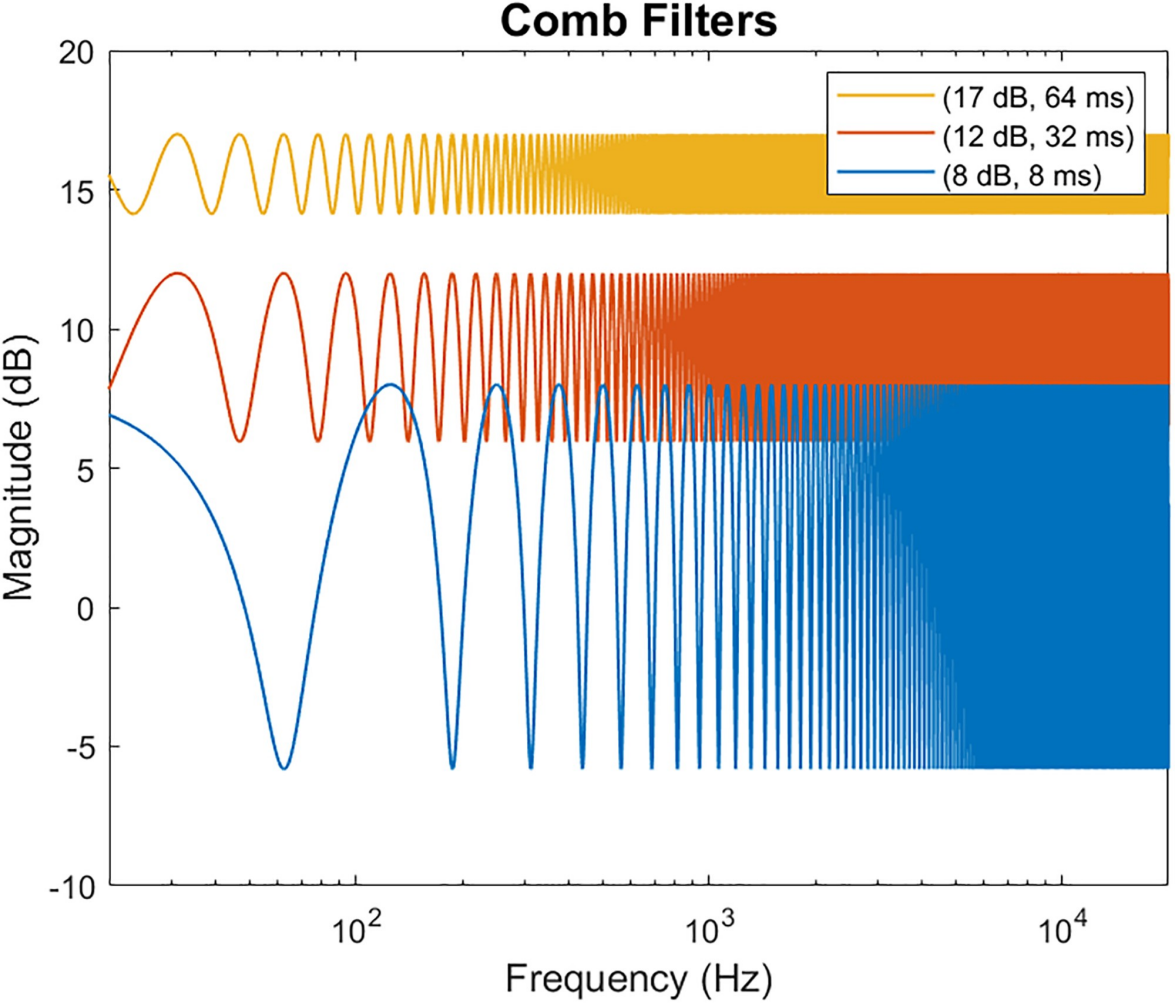
Comb filters. Comb filters as a result of 3 gain / enhancement pairs. Yellow: 17 dB, 64 ms, dip depth = 2.89 dB. Red: 12 dB, 32 ms, dip depth = 6.06 dB. Blue: 8 dB, 8 ms, dip depth = 13.82 dB.

Various amounts of latency between the perception of 2 copies of the same audio signal can result in different types of perceptual effects that may negatively impact the quality of the signal. Studies investigating this phenomenon involve a wide range of stimuli such as clicks, noise bursts, speech, and music. Many of these experiments are often set up such that the lagging stimuli is decreased in intensity to represent an early reflection, in which some of the energy from the direct signal has been lost. The perceptual impacts of latency are heavily dependent on the type of stimuli and the intensity difference between the two occurrences. Latency values on the scale of hundreds of microseconds result in a summing localization where the perceived location varies with amount of delay and level differences [8]. At nearly 1 ms of latency, the two auditory objects become fused into a single auditory object and the summation of the two signals results in constructive and deconstructive interference in periodic amplifications and nullifications across the frequency spectrum [9]. This impact on the frequency spectrum is often referred to as comb filtering and is maximally detectable as reported by a change in sound quality around 1–2 ms [10]. Fig 1 shows the resulting spectrums for the 3 gain / latency pairs used for this study and their resulting comb filter depth. The deeper the notches of the comb filter, the more spectral distortion experienced by the signal. It was speculated that significant amounts of distortion may result in reduced intelligibility. Latency values as little as 5–10 ms have been shown to be perceived as an echo or a separate auditory event [11, 12]. Other studies showed that an echo for speech may not be perceived as a separate event until a lag of over 30 ms (see [13] for review of echo thresholds). When the audio information is compounded with visual information, the latency between audio and visual can persist up to 200 ms, depending on the type of auditory stimulus, before the intelligibility begins to degrade [14].

This entire spectrum of delays and their corresponding perceptual impacts needs to be acknowledged when designing hearing assistive devices that allow the listener to perceive the acoustic and digitally enhanced versions of the signal of interest to the listener. The processing delays found in current digital hearing aids depend heavily on the digital signal processing implemented in the device. Linear phase filters result in constant group delays while non-linear phase leads to a frequency dependent delay. [15] performed a variety of objective measures on 5 different digital hearing aids and found that device time delays vary up to 10 ms. The latency values of the simulated beamformers in this study range from 8 to 64 ms which could result in a range of effects including comb filtering, perceived via coloration of the frequency spectrum, and echo perception, perceived as a second occurrence of the same auditory stimuli. The studies previously mentioned are mostly interested in how the distortion that results from constant group delay impacts sound quality. The purpose of this study was to investigate the relationship between signal enhancement, signal latency, and if the resulting acoustic distortions impact the intelligibility of speech in noise, an important focus for hearing assistive devices. The design of the experiment consists of a simulated hearing environment in which the user perceives two copies of the same speech signal at different gain / latency combinations, where the second copy of the speech is manipulated by a constant group delay, presented in speech shaped noise. The objective of this experiment was to determine the bounds on latency of a beamforming algorithm that would enhance the speech signal in order to improve intelligibility of speech in noise.

## Materials and methods

### Subjects

Thirty-five subjects (19 males) under the age of 55 participated in this study. Four of the subjects were unable to complete the experiment due to technical difficulties. The data shown here is for the remaining 31 participants. Subjects signed a written consent form where they self-reported normal hearing, no neurological deficits, and no formal musical training. The experiment protocol and procedures were approved internally by Facebook Reality Labs’ ethical review board and externally through the Western Institutional Review Board (WIRB).

### Stimulus

**Fig 2:**
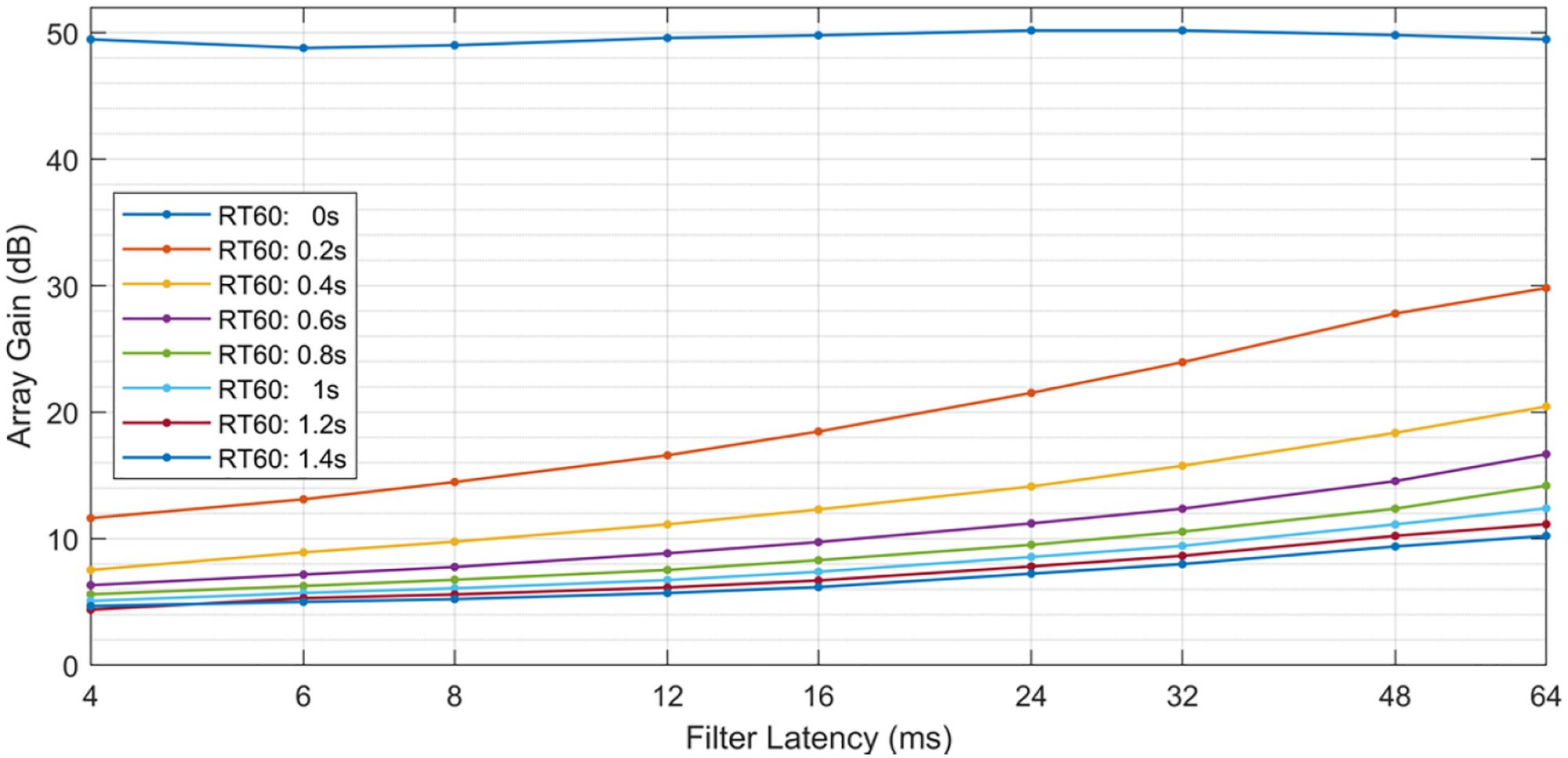
Filter latency vs. array gain. Increased filter length gives larger array gain at the expense of increased latency. Reverberation time by 60 dB (RT60) is given in the legend. (Filter latency = group delay = filter length*0.5) Simulation for one desired source, two interfering sources.

The stimulus used in this experiment comes from the Modified Rhyme Test (MRT). The MRT contains 50 word lists, each containing 6 words that differ in either the first or last consonantal phoneme [16]. The visual presentation of the set of 6 words removes the need to have the listener undergo training of the word set. Traditionally, this set of stimuli is used to test the intelligibility of speech as these signals are transmitted through public safety communication systems. In this study, the MRT was used to determine intelligibility as the speech was transmitted through a simulation of speech enhancement in a noisy environment. The simulation was set up to emulate an open fit hearing aid in which the listener received both the acoustic signal, referred to as the direct path signal, and the output of a simulated beamformer, referred to as the enhanced path signal, with some latency between them. While it would be advantageous to test all combinations of latency and beamformer gain, such an experiment would be prohibitively long for subjects. Instead we focused only on those gain / latency pairs that are likely achievable with real beamformers on actual devices. The gain / latency pairs used in this study were selected based on beamforming simulations and are paired as follows: (8 dB, 8ms), (12 dB, 32 ms), (17 dB, 64 ms). Fig 2 shows the array gain from the beamformer simulations as a function of filter latency (where latency, in samples, of a LTI FIR filter is defined as half of the filter length) for various reverberation times. A value of 0.6s was designated for the reverberation time to reduce by 60 dB (RT60) to reflect a large office room or small lecture room of 200–300 cubic meters [17]. Both the direct and enhanced speech signals were spatialized to 15 degrees azimuth using a generic HRTF to simulate an external talker in front of the listener. This spatialization was used for the listeners to be more likely to externalize the speech signal and perceive it as if the speaker was sitting across from them.

In addition to the presented words, steady-state speech shaped noise (SSN) was also presented. The noise was manipulated to emulate crowd noise by spatializing 8 noise signals in the 8 cardinal directions around the listener using the same generic HRTF set used for the speech stimuli. Each of the 8 noisy signals were uncorrelated in phase to ensure that they did not result in phasing artifacts or summing localization. After spatialization, all speech and noise signals were normalized to 23 loudness units relative to full scale (LUFS) using Adobe’s Audition. This measure of loudness became the experiment’s reference sound level (0 dB). The gain and latency values of the enhanced-to-direct signal were fixed in each adaptive track according to the previously mentioned gain / latency pairs, while the SNR of the enhanced-to-SSN (and as a result, the SNR of the direct-to-SSN) was varied and tracked throughout the experiment. This tracked value, the SNR of the enhanced-to-SSN, was used to calculate the listener’s threshold for correctly perceiving the presented word.

**Fig 3:**
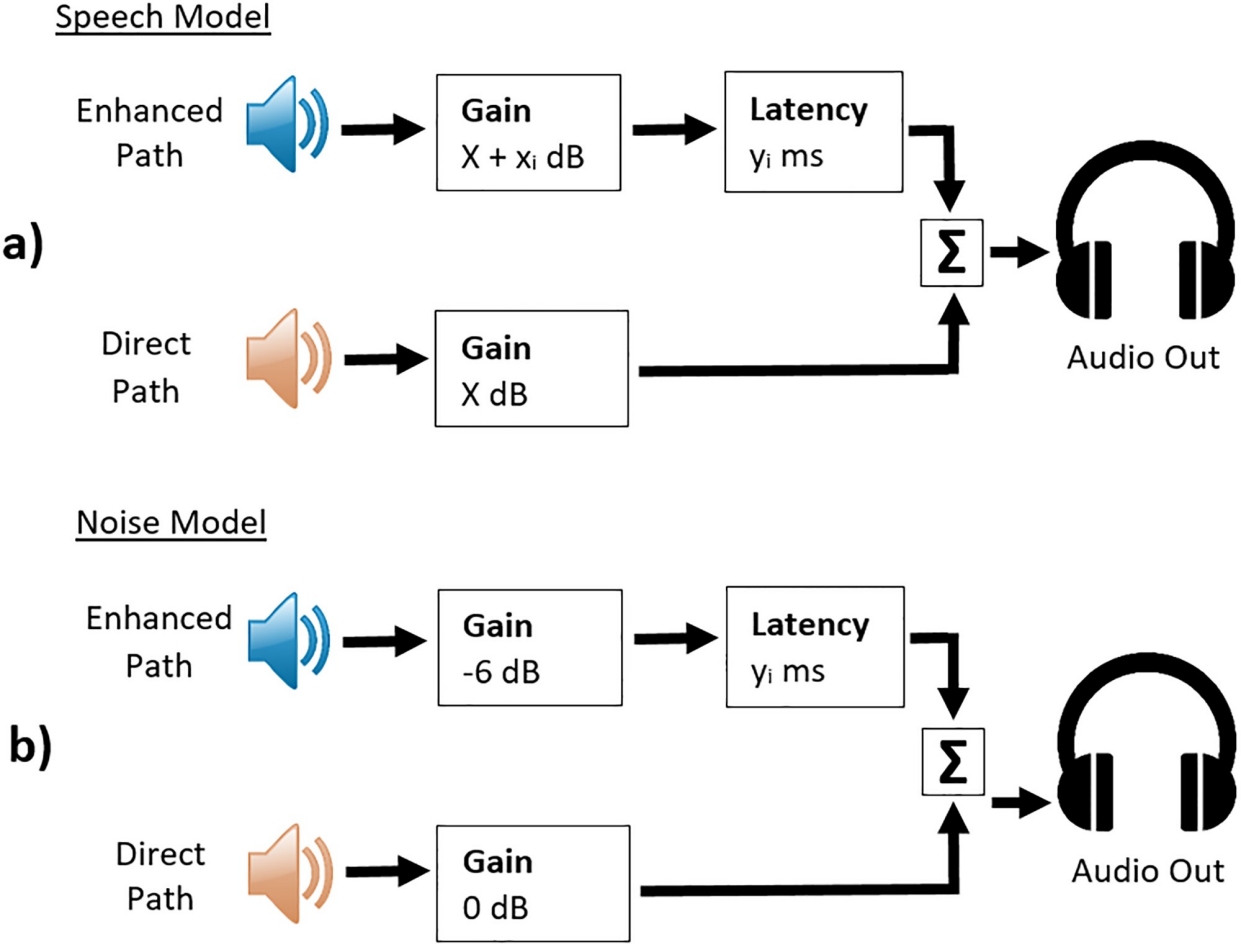
Simplified simulink schematic. a) Speech model containing 2 speech signals–direct path signal set at X dB and varies according to previous subject response. Enhanced path signal contains same speech signal with some additional gain of x_i_ dB and a latency value of y_i_ ms (i = 1,2, or 3; gain / latency pairs are described in text). b) Noise model containing 2 speech shaped noise signals–direct path fixed at 0 db, enhanced path fixed at -6 dB, representing worst case scenario of a successful beamformer, with the same latency value, y_i_, in speech model.

The speech and noise stimuli were presented using MATLAB’s Simulink which allowed for real time manipulation of the signals. The speech and noise were presented in two separate Simulink models and were adjusted in parallel. Each model contained direct and enhanced audio paths. The direct path simulated the acoustic signal from the target speaker, and the enhanced path simulated the amplified digital signal presented to the listener with a pre-determined gain and latency relative to the direct path signal. The noise model’s direct path presented the noise at the 8 cardinal directions at 0 dB, while the enhanced path presented the noise at -6 dB, spatialized to 15 degrees azimuth with the same latency value as in the speech model’s enhanced path. This -6 dB was a relatively arbitrary value assumed to be the worst case noise attenuation of a successful beamformer. The audio signals were sent via Simulink to 2 separate audio inputs in the Babyface Pro sound card. Both of these inputs were routed to the listener’s DT 990 PRO headphones. A simplified schematic of the Simulink setup is described in Fig 3.

### Experiment

**Fig 4:**
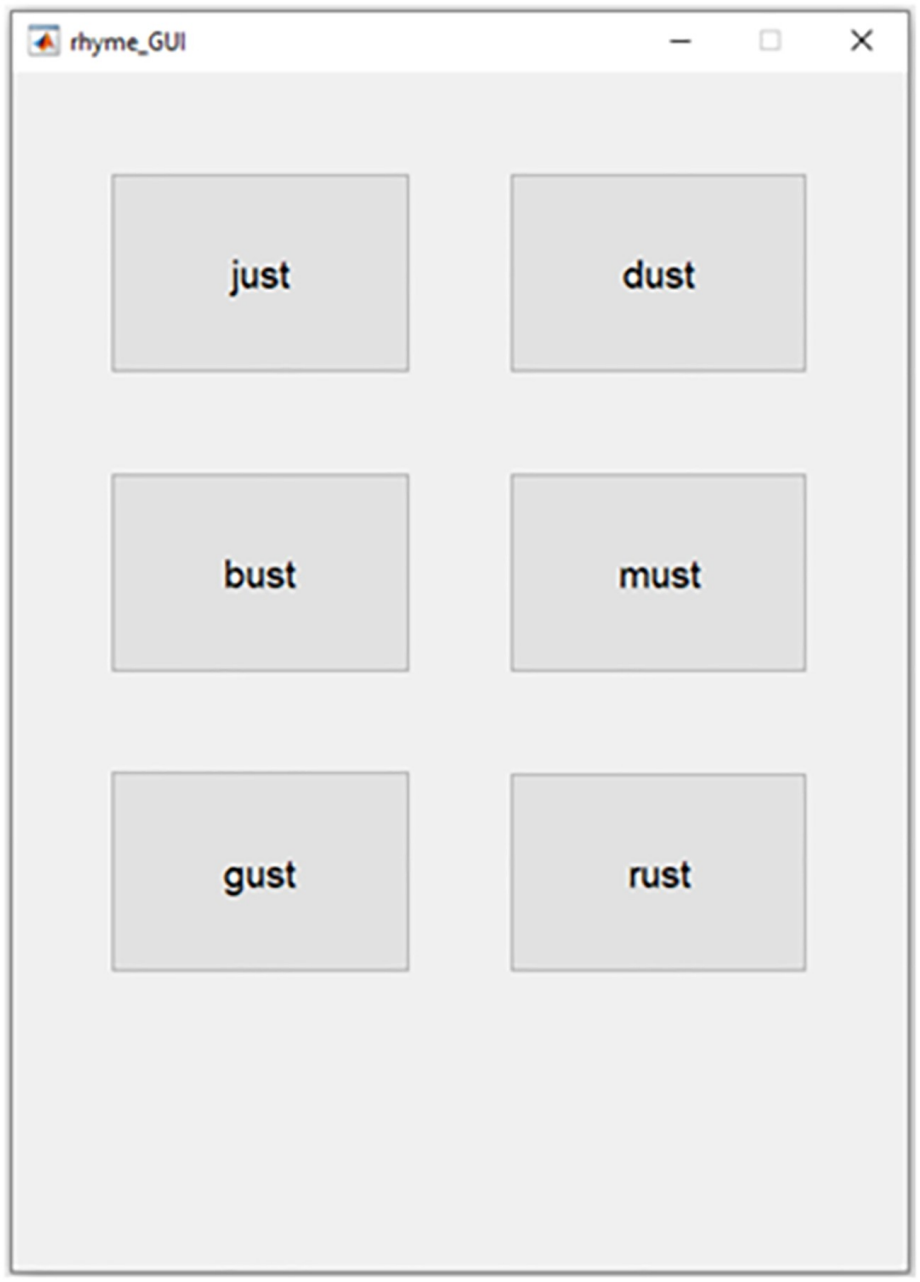
Experiment’s guided user interface. Example of MATLAB’s GUI for a single trial of implementing the Modified Rhyme Test (MRT).

This experiment incorporated an interleaved adaptive track using a 2-down-1-up rule converging at 8 reversals to predict a 70.7% likelihood of correct word understanding [18]. The speech intelligibility threshold was calculated using the last 5 reversals for each adaptive track. Each participant completed 4 adaptive tracks per condition for a total of 12 adaptive tracks each resulting in their own threshold value. The adaptive tracks were delivered in 3 blocks of 4 tracks each. Each block randomly selected which tracks, corresponding to which conditions, were presented to the subject. Throughout the experiment, while the gain and latency between the direct and enhanced signals were fixed, the SNR of the enhanced signal relative to the SSN was varied. The objective of the experiment was to determine the SNR threshold in which speech could be understood in steady-state SSN. This experiment also tested whether latency between two copies of the same speech signal would impact speech intelligibility. The latency value of the enhanced path for speech and the enhanced path for noise, as well as the gain values of the speech model were all adjusted in response to the user’s most recent selection, per adaptive track. Using MATLAB’s graphical user interface, the subject was prompted to select 1 of 6 words that appeared on the screen (Fig 4). Each block lasted approximately 10 minutes. At the end of each block, the subject was given the opportunity to take a short break.

### Data acquisition

Using MATLAB’s graphical user interface, the subject was prompted to select the word presented in the stimulus. The presented stimulus, subject’s response, and correctness of the response were recorded in separate arrays. Once 8 reversals were achieved, the last 5 reversals were used to calculate a mean threshold per track. Post processing consisted of calculating the mean threshold per simulation condition per listener.

### Intelligibility

In order to quantify the simulation’s impact on intelligibility with the variation of gain / latency across the 3 conditions, the Hearing Aid Speech Perception Index (HASPI) [19] was implemented. The HASPI model was left unmanipulated to resemble a healthy auditory system. Additionally, the short-time objective intelligibility (STOI) [20] measure was also utilized to further support the psychoacoustic results. The reference signal for both metrics was a clean speech signal, while the test signal contained the combination of the enhanced signal at a specified latency and gain, the direct signal, and the speech shaped noise.

## Results

This experiment used a simulated beamformer to test whether the perceptual impacts from latency would outweigh the benefits from an increase in SNR. The simulation contained 3 signals: direct path signal, simulating the acoustic signal from the speaker to the listener’s ear, the enhanced path signal, simulating the digitally enhanced signal from the device to the listener’s ear, and the speech shaped noise, simulating the background noise. These 3 signals were combined in 3 combinations: the gain of the enhanced to direct signal was fixed at 8, 12, or 17 dB. These 3 gains were accompanied by a latency of 8, 32, and 64 ms respectively. The variable of interest to this study was the SNR of the enhanced signal relative to the SSN. It was hypothesized that as the latency increased, the intelligibility of the speech signal would decrease.

**Fig 5:**
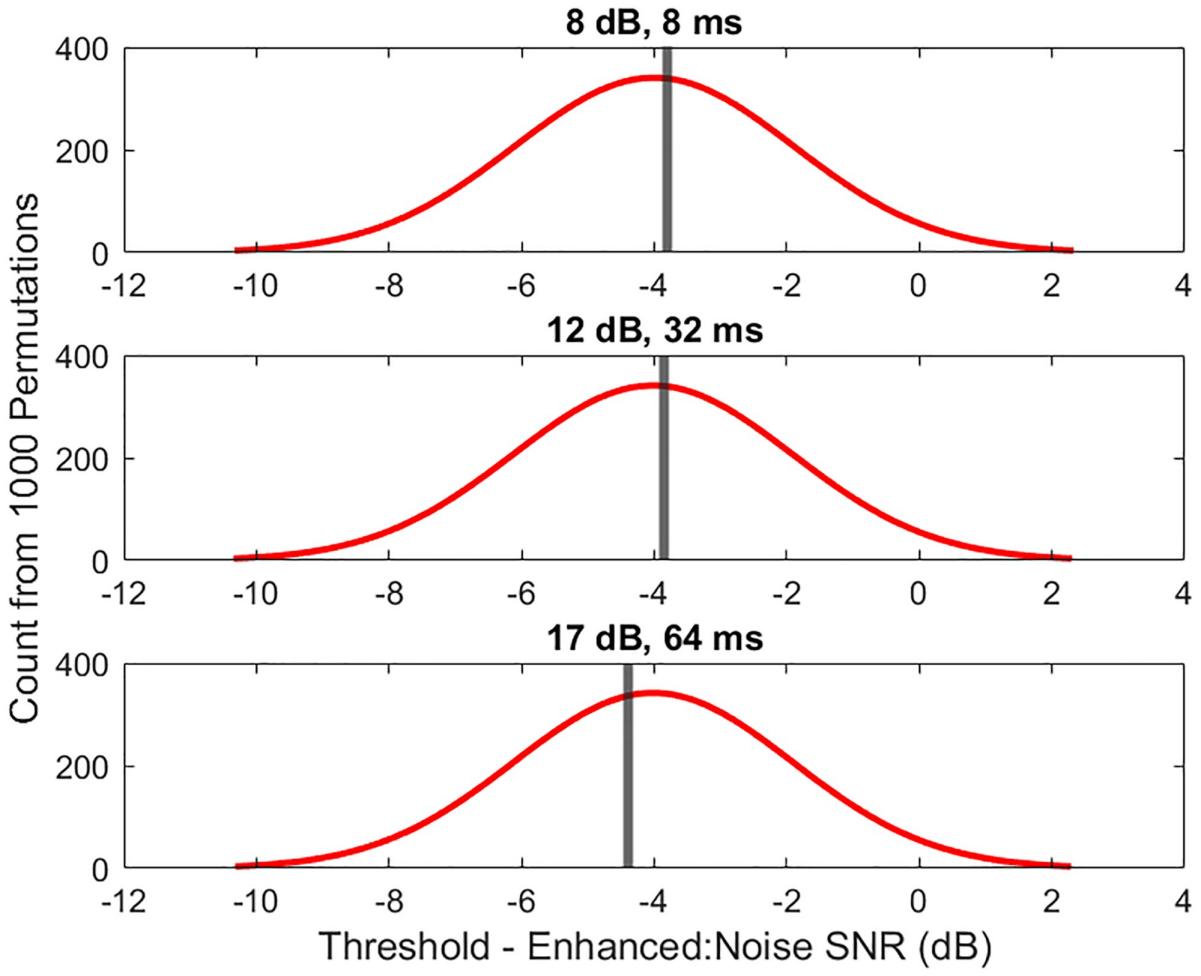
Visual for permutation statistics. The distribution of thresholds from the permutation statistics is shown in red. The gray line shows where the average from the experimental data falls within the distribution.

The results of the experiments are in support of the null hypothesis–an increase in latency between the enhanced and direct speech signals does not have a negative impact on the intelligibility of the speech signal, with the caveat that the direct signal is masked by noise. Table 1 shows the average SNR of the enhanced signal to noise was not significantly different across conditions. It is worth noting here that the SNR of the direct signal to noise was different across conditions, although this is not the value we are tracking in this experiment. It could be argued that these differences in SNR of the direct signal to noise mean that there are notable differences in intelligibility across conditions, but for the sake of consistency we are only focusing on the SNR of the enhanced signal to noise in this article. The individual data points behind the values reported in Table 1 are reported as S1 Table. In order to confirm this result, permutation statistics were computed using 1000 iterations of randomly shuffling the 3 condition labels of the average thresholds per condition per subject (31 subjects * 3 conditions = 93 total thresholds), and recalculating the averages per conditions. These 1000 averages per condition were then averaged, and the standard deviation calculated. Fig 5 shows the distribution of SNRs of the permutation statistics in red. The gray line shows where the recorded data’s average values, calculated from thresholds obtained through the experimental protocol, lie as compared to the distribution of permutation statistics. As expected, the experimental averages nearly align with the maximum value of the permutation statistic’s distribution, confirming insignificant differences across conditions.

**Table 1:** Experimental conditions and threshold results. Three gain / latency combinations from the RT60 = 0.6s beamforming simulation from Fig 2 used in this experiment. The average thresholds (SNR of enhanced signal to noise) and standard deviation across subjects for each of the 3 conditions. (dB = decibels; ms = milliseconds).

Array Gain (dB)	Latency (ms)	Average SNR (dB)	Standard Deviation
8	8	-3.80	1.99
12	32	-3.85	2.24
17	64	-4.39	2.03

**Fig 6:**
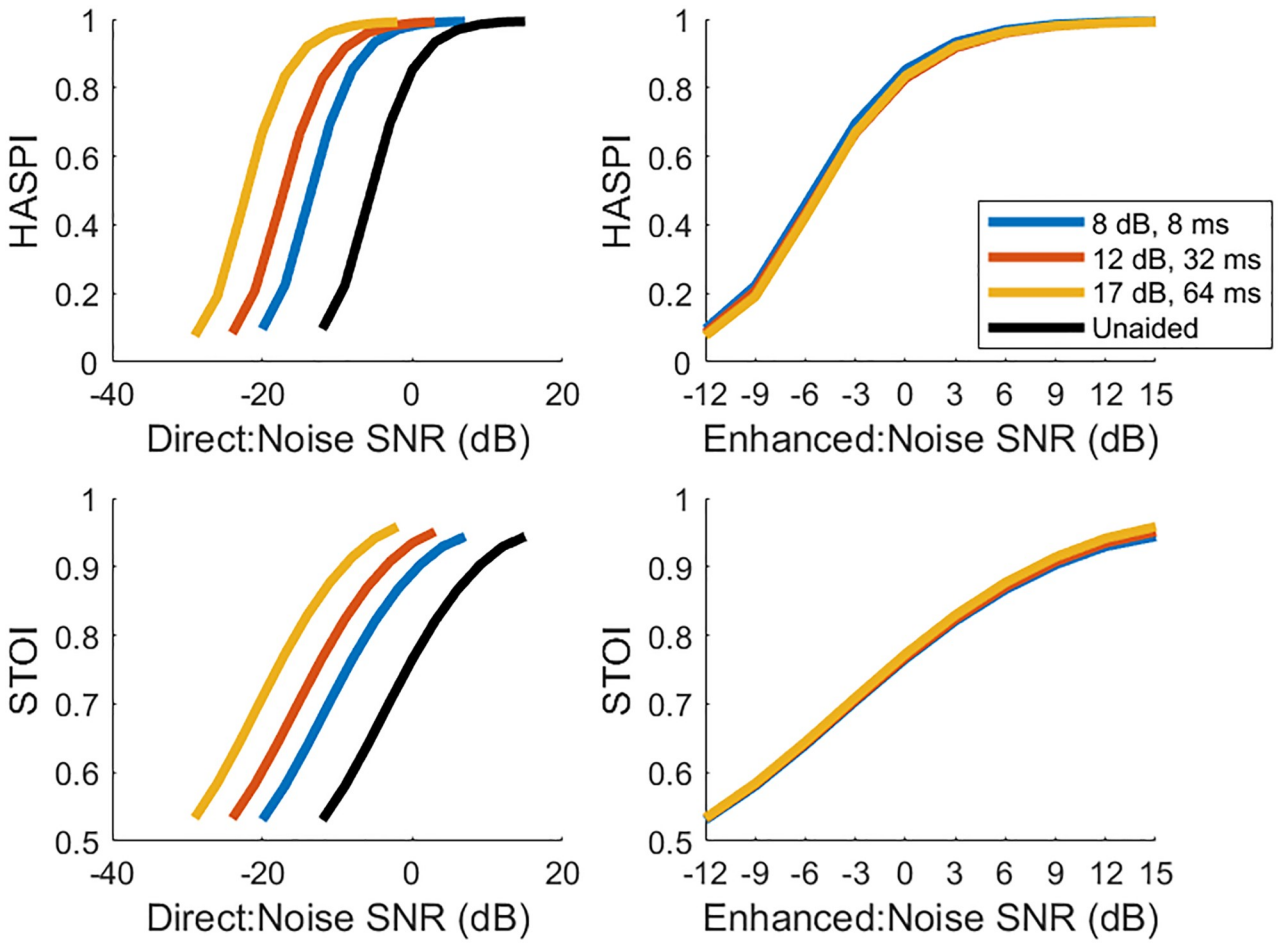
Intelligibility metrics. Left graphs show the HASPI and STOI (top and bottom, respectively) as a function of the direct path signal-to-noise ratio. Adding the various enhancements translates the curve by the corresponding amount of gain. Right graphs show the HASPI and STOI (top and bottom, respectively) as a function of the enhanced path signal-to-noise ratio. Regardless of the direct path signal level, the enhanced signal dominates the intelligibility calculation.

To further support these results, the HASPI and STOI metrics were used in order to quantify the impact that the simulation had on the intelligibility of the speech signals. The three conditions show a very similar monotonic increase in intelligibility as the SNR increases, with no significant difference across conditions for both metrics. Fig 6 (left) shows the HASPI and STOI (top and bottom, respectively) values as a function of the direct path SNR. The figure shows that the larger the enhancement or gain, the less SNR for the direct signal is needed for intelligibility. Fig 6 (right) shows the HASPI and STOI (top and bottom, respectively) values as a function of the enhanced path SNR. This figure shows that the intelligibility is nearly the same across the three conditions. This informs us that the enhanced signal is the dominating signal when the direct signal is masked by noise, meaning listeners primarily use the enhanced signal to understand the message.

## Discussion

The results of this study show no significant difference in intelligibility between the 3 gain / latency conditions. For this simulation, there was no significant relationship between the SNR of the enhanced signal to noise, or the latency between speech signals, and the perceptual thresholds of speech intelligibility. As noted in the results section, there are differences in the SNR of the direct signal to noise, but this article is focused on the SNR of the enhanced signal to noise. The average SNR threshold of understanding between the enhanced signal and the noise was -4.01 +/- 0.27 dB across conditions. In order to confirm the conclusion that there were no significant differences across conditions, permutation statistics were computed. By randomly permuting the labels (representing which condition was tested) of each threshold values, recalculating the averages per conditions, and taking the standard deviation of these new averages, it is shown that the results lie well within the standard deviation of these permutations (see Results for details on calculation). This confirms that the 3 conditions studied here have no significant difference in intelligibility. These results lead us to the conclusion that the amplitude of the enhanced signal was the primary factor in intelligibility in this simulation. The intelligibility metrics used here, HASPI and STOI, confirm the perceptual results, showing insignificant differences in intelligibility scores across the 3 conditions.

**Fig 7:**
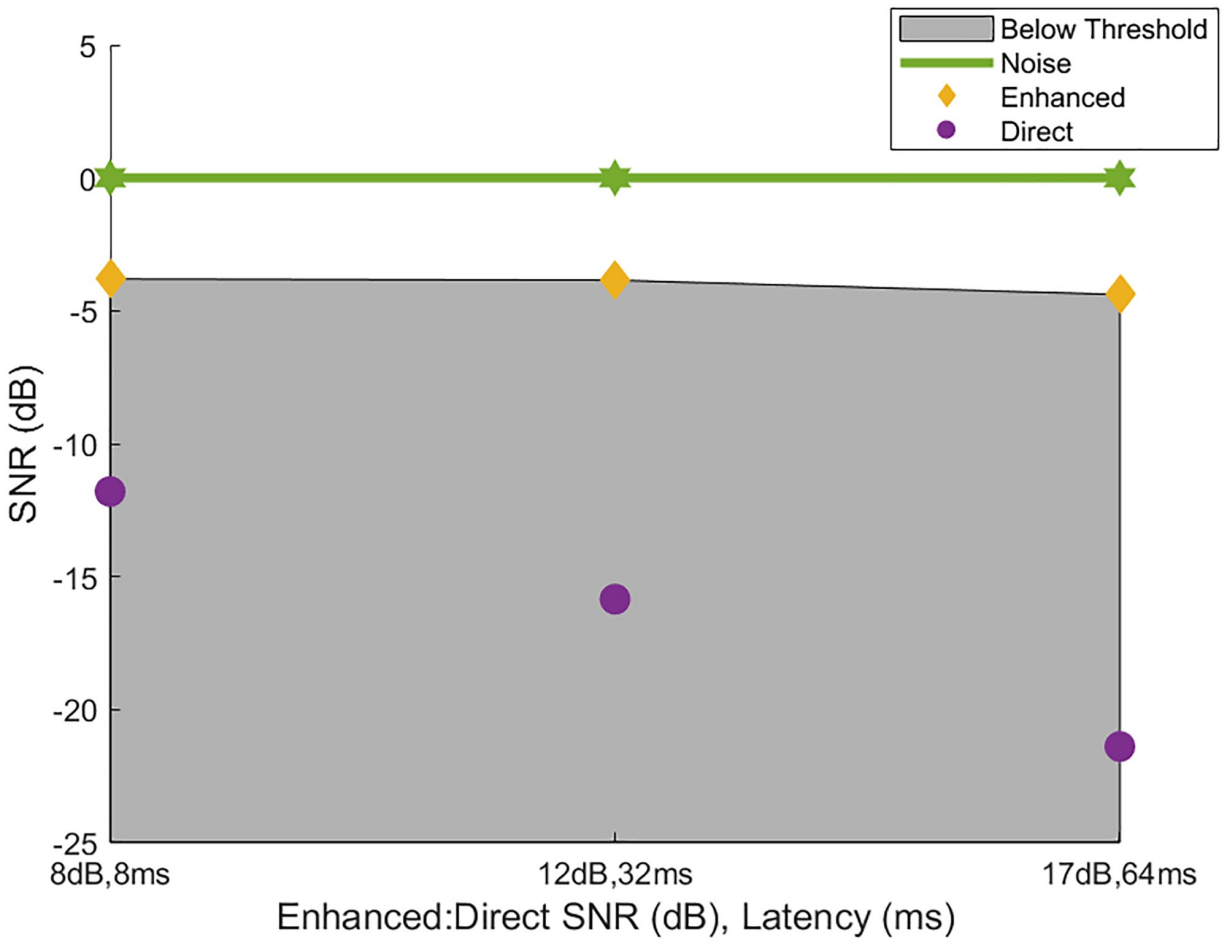
Signal SNR across conditions. Green line is noise level across conditions. Gray region shows the levels below the measured speech intelligibility threshold by the average listener. Yellow diamonds represent the threshold levels across conditions for the average listener. Purple circles indicate level of direct signal, unintelligible and in need of enhancement.

The original hypothesis for this study was that an increase in latency would have a negative impact on the intelligibility of a speech signal. While this may be true for the combination of an enhanced and direct signal in quiet, in which perceptual impacts would be very apparent, it does not apply to the use case of interest. In application, signal enhancement would only be necessary in challenging listening situations, such as a noisy restaurant or cafe, in which a gain of an omnidirectional microphone would not suffice in improving intelligibility and/or SNR of a target speaker. In this situation, the direct signal is masked by the noise, and masked with it are any negative perceptual impacts from latency, while the enhanced signal is the primary signal used for understanding. In the most extreme condition with a latency of 64 ms, there is a noticeable echo, or second occurrence of the same auditory signal (irrelevant of relative amplitudes). However, the results show that this echo does not have a significant impact on intelligibility. Fig 7 shows a visual representation of how the 3 signals presented in the simulation interact with the perception of the listener. The green line shows the noise level across conditions. All stimuli were adjusted according to this noise level, i.e. the noise is set to 0 dB. The gray box shows where speech level is below threshold for the average listener as determined by the experimental results. The yellow diamonds represent the thresholds per condition, indicating the level in which the average listener would be able to correctly identify the word spoken 70.7% of the time (see experimental methods for details). The purple circles represent the direct signal, which are unintelligible and in need of enhancement. This figure shows that regardless of the SNR of the direct signal and latency between the two speech signals, as long as the enhanced signal was greater than about -4 dB, the signal was intelligible.

It should be noted here that the delayed signals in this study are greater in amplitude than the preceding stimuli. This is on the contrary to most studies on this topic in which the delayed signal is lesser in amplitude compared to the preceding signal. Although outside the scope of this study, this brings up the question of symmetry—the order of presenting copies of an auditory stimuli and what types of distortion arise when the first or second presentation is higher in amplitude. This question, in addition to a quantitative measure of sound quality are probable aims for future directions of this research. Majority of the studies in reference here are seeking to understand how early reflections distort the quality of the signal and design their stimuli accordingly. This study is inherently different as it sought to understand how the delivery of an enhanced and delayed copy of an acoustic stimuli, when both the enhanced and acoustic stimuli are perceived, would impact the intelligibility of that stimuli (see Methods for stimuli details). In regards to speech, a decrement in quality relates to tradeoffs between audibility and distortions [21] whereas a change in intelligibility is primarily focused on the audibility of the signal, often correlated with changes in envelope and temporal fine structure [19].

Although our simulation may not completely encapsulate the dynamics of a real-world cocktail-party scenario, the results of this experiment provide an important finding for the development of hearing assistive devices that implement beamforming. The original thought was that latency would have to be constricted to less than 2 ms to avoid distortions such as comb filtering that may negatively impact the intelligibility of the signal. The results show that, when the direct signal is masked by the noise and unintelligible, the negative impacts on intelligibility from short latency values are also masked. While extreme changes in coloration can impact speech understanding, low-level coloration effects here were not associated with a change in intelligibility. In particular, coloration from comb filtering is not perceived in this scenario because the first of two signals is masked and does not interact with the second signal in the manner necessary to produce said effect at a high enough level to impact intelligibility. This is especially true for the hearing impaired community, where the direct signal is likely to be unintelligible and/or unperceived. Therefore, in developing open ear hearing assistive devices that utilize beamforming, the designers need to be less worried on latency constraints from digital signal processing algorithms, and more concerned with distractions that may arise from perceiving one’s own voice, as well as audio-visual mismatch. A distorted perception of one’s own voice can happen anywhere between 2 and 50 ms (see [22] for a review). This provides a strict constraint of processing delay and would likely need creative solutions to work around. One possibility being the implementation of a self-voice detection system utilized such that when the user of the device is speaking, the enhancement is turned off so as to not amplify the user’s voice at all. The other constraint here would be the audio-visual (AV) mismatch in which the listener hears the speech after a noticeable delay from seeing the speaker’s mouth produce the words. For speech signals this delay can be up to 200 ms before negatively impacting intelligibility [14] which provides plenty of time for implementing a beamformer alone. However, if quality or naturalness of the perceived speech is the primary objective over intelligibility, which is beyond the scope of this study, then the constraints on latency may be much more stringent. [23] determined that latency values around 4 ms were perceived as alterations in sound quality when subjects listened to their own voice through a DSP hearing aid. Future studies may investigate signal processing schemes that can preserve the sound quality for realistic latency values in beamforming algorithms, similar to those used in this study.

In conclusion, this study weighed the benefits of providing an enhanced or amplified signal versus the costs of latency that inherently accompany the signal processing performed to provide the enhanced signal. The results of this study show that with enough gain, the latency shows no significant impact on intelligibility when the signals are presented in noise. This finding greatly reduces the constraints on this problem in realistic use cases where a listener is having trouble hearing a target speaker in a noisy environment. While an appropriate gain does outweigh the impacts of latency, there are still realistic constraints regarding AV mismatch. Even with an appropriate gain, if the latency exceeds 200 ms, the intelligibility is likely to be negatively impacted if the user sees the talker speaking and hears the talker’s words at different instances of time.

## Supporting information

S1 Table Thresholds per trial. Thresholds of enhanced-to-SSN SNR for each of our 31 subjects across 12 trials– 4 trials per condition.
(DOCX)
