# Supplementary material for: Negative impacts from latency masked by noise in simulated beamforming
Source: PLoS One. 2021 Jul 1;16(7):e0254119. doi: 10.1371/journal.pone.0254119 (PMC8248715; doi:10.1371/journal.pone.0254119)
Supplement: S1 Table — Thresholds of enhanced-to-SSN SNR for each of our 31 subjects across 12 trials– 4 trials per condition. (DOCX) [file pone.0254119.s001.docx]

|  | **(8 dB, 8 ms)** | | | | **(12 dB, 32 ms)** | | | | **(17 dB, 64 ms)** | | | |
| --- | --- | --- | --- | --- | --- | --- | --- | --- | --- | --- | --- | --- |
| **Subj 1** | -6.00 | -1.91 | -1.00 | -5.00 | -5.36 | -8.55 | -6.57 | 0.69 | -6.86 | -6.00 | -3.60 | -2.40 |
| **Subj 2** | -1.40 | -1.42 | -2.60 | -3.00 | 0.92 | -9.46 | -5.40 | -5.00 | -3.86 | -5.45 | -7.25 | -4.62 |
| **Subj 3** | -6.55 | -4.83 | -3.92 | -1.20 | -1.13 | -7.80 | -6.18 | -9.23 | -2.73 | -3.69 | -9.60 | -3.60 |
| **Subj 4** | -4.57 | -4.40 | -2.70 | -8.00 | -1.15 | -6.86 | -5.45 | -6.00 | -2.40 | -6.40 | -7.13 | -3.33 |
| **Subj 5** | -3.82 | -5.00 | -1.67 | -3.14 | 8.25 | -8.12 | -1.63 | -2.36 | -4.75 | -1.62 | -7.80 | -6.53 |
| **Subj 6** | -8.18 | 0.25 | -9.46 | -4.89 | -2.82 | -7.00 | -10.09 | -3.80 | 0.90 | -1.85 | -7.13 | -1.07 |
| **Subj 7** | -1.13 | -4.75 | -3.69 | -4.74 | 3.00 | 3.67 | -2.80 | 3.80 | -7.40 | -1.13 | 3.00 | -4.64 |
| **Subj 8** | -8.33 | -5.75 | -6.47 | -4.13 | -0.86 | 1.00 | -10.00 | -3.33 | -1.71 | -3.60 | -1.06 | -4.83 |
| **Subj 9** | 3.27 | -2.60 | -4.15 | 0.57 | -7.85 | 0.00 | -3.00 | -5.57 | 0.21 | -9.20 | -3.55 | -2.25 |
| **Subj 10** | 5.47 | -3.33 | 4.29 | -1.91 | -9.82 | -2.70 | 6.67 | 0.75 | 4.24 | -4.71 | -2.44 | -2.70 |
| **Subj 11** | -4.25 | -0.25 | -5.45 | -3.56 | 1.75 | -2.60 | 0.25 | -5.45 | -5.08 | -3.33 | -5.45 | -3.46 |
| **Subj 12** | 0.92 | 2.73 | -5.00 | 2.50 | 3.67 | 6.00 | 2.10 | -7.13 | -1.25 | -0.88 | 3.23 | -2.33 |
| **Subj 13** | -7.33 | -6.23 | -8.21 | -5.50 | -2.68 | -7.13 | -3.64 | -5.40 | -2.40 | -1.91 | -9.82 | -5.75 |
| **Subj 14** | -4.15 | -7.00 | -6.63 | -6.86 | -5.31 | -6.50 | -7.13 | -4.33 | -6.14 | -4.62 | -7.31 | -4.94 |
| **Subj 15** | -6.50 | -1.31 | -4.50 | -10.50 | -6.00 | -1.13 | -7.91 | -0.86 | -1.41 | -5.14 | -8.18 | -7.13 |
| **Subj 16** | -3.75 | 0.40 | -2.40 | -7.13 | -4.00 | -4.05 | -2.33 | -9.00 | -2.25 | -7.43 | -1.35 | -10.64 |
| **Subj 17** | -3.00 | -2.40 | -4.31 | -7.75 | -5.31 | -4.31 | -3.82 | -9.33 | -4.64 | -7.09 | -2.81 | -7.13 |
| **Subj 18** | -7.07 | 3.00 | 0.27 | -9.33 | 0.92 | -6.23 | -9.21 | -1.71 | -5.31 | -4.29 | -4.64 | -3.69 |
| **Subj 19** | -2.06 | 0.40 | 2.18 | 4.38 | 10.88 | -1.76 | -5.25 | -7.13 | 6.50 | -1.38 | 2.77 | -6.00 |
| **Subj 20** | -9.19 | -3.20 | 0.56 | -1.58 | 3.00 | -7.74 | -0.86 | -5.00 | -2.57 | -6.94 | -1.75 | -4.57 |
| **Subj 21** | -7.33 | -2.65 | -7.15 | -5.36 | -6.00 | -3.33 | 2.00 | -2.40 | -8.36 | 0.60 | -7.20 | -1.06 |
| **Subj 22** | -3.33 | -0.38 | -4.15 | -8.57 | -9.00 | -7.56 | -1.91 | -2.17 | -6.46 | -6.25 | -7.13 | -1.60 |
| **Subj 23** | -5.67 | -5.36 | -5.75 | -2.73 | -9.00 | -1.94 | -4.64 | 0.64 | -1.38 | -1.91 | -2.82 | 0.00 |
| **Subj 24** | -5.75 | -0.86 | -3.94 | -6.33 | -9.46 | -2.36 | -6.29 | -7.13 | -6.86 | -4.00 | -8.04 | -10.88 |
| **Subj 25** | -5.14 | -6.00 | -1.33 | -6.95 | -8.75 | -7.20 | -3.86 | -5.45 | -4.91 | -5.33 | -5.40 | -1.13 |
| **Subj 26** | -0.20 | -6.19 | -3.82 | -3.00 | -2.00 | -8.70 | -3.00 | -4.35 | 4.11 | -9.21 | 0.25 | -2.25 |
| **Subj 27** | -3.56 | -10.24 | -5.31 | -6.50 | -6.75 | 0.00 | -9.00 | -9.56 | -10.38 | -8.70 | -5.45 | -8.73 |
| **Subj 28** | -9.33 | 2.25 | -5.53 | -7.13 | -3.86 | -2.70 | -10.25 | -6.00 | -8.06 | -9.33 | -7.13 | -3.00 |
| **Subj 29** | -5.75 | -0.56 | -2.33 | -7.20 | -2.08 | -1.71 | 6.80 | -5.31 | -5.10 | -11.33 | -6.86 | -0.15 |
| **Subj 30** | -6.00 | -3.55 | -1.13 | -3.82 | -4.15 | -1.33 | -9.94 | -3.00 | -8.18 | -3.33 | -6.86 | -10.09 |
| **Subj 31** | -5.79 | 1.75 | -9.00 | -3.60 | -2.36 | -9.00 | -7.13 | -7.15 | -5.40 | -9.69 | -7.07 | -3.00 |

**S1 Table. Thresholds per trial.** Thresholds of enhanced-to-SSN SNR for each of our 31 subjects across 12 trials – 4 trials per condition.
